# FISH^+^CD34^+^CD38^-^ cells detected in newly diagnosed acute myeloid leukemia patients can predict the clinical outcome

**DOI:** 10.1186/1756-8722-6-85

**Published:** 2013-11-07

**Authors:** Libing Wang, Lei Gao, Sheng Xu, Shenglan Gong, Li Chen, Shuqing Lü, Jie Chen, Huiying Qiu, Xiaoqian Xu, Xiong Ni, Xianmin Song, Weiping Zhang, Jianmin Yang, Min Liu, Xiaoxia Hu, Jianmin Wang

**Affiliations:** 1 Institute of Hematology, Changhai Hospital, Second Military Medical University, Shanghai 200433, China

**Keywords:** Acute myeloid leukemia, Leukemia initiating cells, Minimal residual disease

## Abstract

**Background:**

In acute myeloid leukemia (AML), the leukemia initiating cells (LICs) or leukemia stem cells (LSCs) is found within the CD34^+^CD38^-^ cell compartment. The LICs subpopulation survives chemotherapy and is most probable the cause of minimal residual disease (MRD), which in turn is thought to cause relapse. The aim of this study was to determine the prognostic value of the percentage of LICs in blasts at diagnosis.

**Design and methods:**

The percentage of LICs in the blast population was determined at diagnosis using a unique Flow-FISH analysis, which applies fluorescent in situ hybridization (FISH) analysis on flow cytometry sorted cells to distinguish LICs within the CD34^+^CD38^-^ cell compartment. Fourty-five AML patients with FISH-detectable cytogenetic abnormalities treated with standardized treatment program were retrospectively included in the study. Correlations with overall survival (OS), events-free survival (EFS) and cumulative incidence of relapse (CIR) were evaluated with univariate and multivariate analysis.

**Results:**

The percentage of LICs is highly variable in patients with acute myeloid leukemia, ranged from 0.01% to 52.8% (median, 2.1%). High LIC load (≥1%) negatively affected overall survival (2-year OS: 72.57% vs. 16.75%; *P* = 0.0037) and events-free survival (2-year EFS: 67.23% vs. 16.33%; *P* = 0.0018), which was due to an increased cumulative incidence of relapse (2-year CIR: 56.7% vs. 18.0%; P = 0.021). By multivariate analysis, high LIC load retained prognostic significance for OS and EFS.

**Conclusions:**

In the present study, we established the Flow-FISH protocol as a useful method to distinguish normal and leukemic cells within the CD34^+^CD38^-^ cell subpopulation. The high percentage of LICs at diagnosis was significantly correlated with increased risk of poor clinical outcome.

## Background

In acute myeloid leukemia (AML), induction therapy consisting of an anthracycline and cytarabine is still considered to be the gold standard regimen for younger patients, resulting in complete remission rates of 50 to 75% [1]. However, relapse is common and the 5-year overall survival remains less than 40% even after high-dose chemotherapy and stem cell transplantation [2]. Relapse likely originates from minimal residual disease (MRD) cells, which can be detected after chemotherapy. Consequently, quantifying MRD in AML, using flow cytometery or molecular procedures, is of prognostic importance: MRD frequency after chemotherapy strongly correlates with the incidence of relapse and survival [3-5].

Acute myeloid leukemia (AML) is a clonal hematologic malignancy arising from a small population of leukemic cells that initiate and propagate the disease [6,7]. These cells are termed leukemic stem cells (LSCs) or leukemia initiating cells (LICs), which are derived from normal hematopoietic stem cells or from more mature myeloid progenitors. LICs are resistant to chemotherapeutic agents and therefore likely responsible for the outgrowth of MRD, which in turn is thought to cause relapse [8]. Since in this concept the LICs are regarded as the root of MRD and relapse, detection of leukemia initiating cells at diagnosis might offer prognostic value in predicting relapse of the disease. However, LICs appear to share similar cell phenotypic characteristics previously identified for normal HSCs, such as CD34 and CD38, several groups have reported that some markers are differentially expressed between the two such as CD90, CLL-1 and IL-3 receptor [9-11]. It is still difficult to distinguish LICs from their normal counterparts by flow cytometery precisely.

Previous studies have shown that the enrichment of the CD34^+^ or CD34^+^CD38^-^ phenotype in AML at diagnosis were associated with a high level of residual disease after treatment [12,13]. In the present study, we established a unique protocol for applying fluorescent in situ hybridization (FISH) analysis on Fluorescence-activated cell sorting sorted cells, and separated LICs and normal HSCs within the CD34^+^CD38^-^ cell compartment of AML patients at diagnosis. Our study found that percentage of FISH-positive CD34^+^CD38^-^ cells greater than 1% was strongly correlated with decreases in both events-free and overall survival in AML patients.

## Methods

### Patients

In this study we included 45 *de novo* AML patients (25 male, 20 female) with a median age of 42 years (range, 14–71), admitted in the Institute of Hematology, Changhai hospital, between June 2010 and October 2012 (Table 1). Patients with a history of myelodysplastic syndrome or therapy-related AML as well as acute promyelocytic leukemia were not included. The patients had the following cytogenetic abnormalities: t(8; 21) (n = 22), inv(16) (n = 6), trisomy 8 (n = 5), *MLL* rearrangement (n = 3), trisomy 21 (n = 2), 7q^-^ (n = 2), 9q^-^ (n = 2) and t(6;9) (n = 1). (Additional file 1: Table S1) All research samples represented excess bone marrow collected at diagnosis. The present study was approved by the Changhai Hospital Institutional Review Board and signed informed consent was obtained from each patient in accordance with the Declaration of Helsinki.

**Table 1:** Characteristics of the 45 AML patients at diagnosis

	**All patients**	**FISH + CD34 + CD38-**	**FISH + CD34 + CD38-**	***P *** **value**
	**N = 45**	**<1%**	**≥1%**	
		**N = 20**	**N = 25**	
Gender				
Male(%)	25(55.6)	10(50)	15(60)	0.557
Female(%)	20(44.4)	10(50)	10(40)	
Age, Median(range)	42(14–71)	36(19–71)	45(14–66)	0.5600
WBC, Median(range)	14.5(0.63-143)	12.4(0.63-125.24)	14.9(1.7-143)	0.9091
PLT, Median(range)	42(4–191)	28(4–191)	48(16–157)	0.198
BM blast%	58(18–96)	52.5(20–94.5)	62(18–96)	0.265
FAB subtype				0.694
M1	5	2	3	
M2	18	10	8	
M4	12	5	7	
M5	9	3	6	
M6	1	0	1	
Cytogenetics risk				0.894
Favorable	24	10	14	
Intermediate	12	6	6	
Poor	9	4	5	
FLT3 mutations (%)	5(11.1)	2(10)	3(12)	1
Allo-HSCT(%)	14(31.1)	6(30)	8(32)	1

Patients were stratified into favorable (cases with t(8;21), or inv(16), excluding complex cytogenetic), poor (cases with complex cytogenetic changes three or more unrelated abnormalities, t(6;9) or 11q23 abnormalities [excluding t(9;11)]), and intermediate (cases with t(9;11), trisomy 8, 7q-, 9q-, or trisomy 21) risk groups.

### Treatments

Induction treatment comprised Daunorubicin (DNR) at 45–60 mg/m^2^/d or Idarubicin (IDA) at 8-10 mg/m^2^/d by 30-minute IV infusion on days 1 to 3 and Cytarabine (Ara-C) at 100 mg/m^2^/d by continuous IV infusion from days 1 to 7 (DA regimen). In patients not reaching CR after 2 courses of DA, a salvage course (FLAG), consisted of five days of treatment with a 30-minute infusion of fludarabine (Flu) 30 mg/m^2^/d followed, four hours later, by a 4-hour infusion of Ara-C 2 g/m^2^/d, was given. Granulocyte colony-stimulating factor (G-CSF) 300 μg/day s.c. was administered 12 hours before starting fludarabine, for five days and then continued after the end of therapy until myeloid recovery. Patients reaching CR then received 4 monthly consolidation cycles with Ara-C at 2 g/m^2^/12 h by 2-hour IV infusion for 3 days, followed by G-CSF starting at day 8 or neutrophile counts less than 1 × 10^9^/L until neutrophil recovery. Patients with CNS disease received triple intrathecal infusions (methotrexate 10 mg, cytarabine 50 mg, dexamethasone 5 mg) twice a week until disappearance of blast cells in the cerebrospinal fluid. Patients were candidates for allogeneic hematopoietic stem cell transplantation (allo-HSCT) in first CR if they had a matched sibling or ≥9/10 HLA allele fully matched unrelated donor. Standard myeloablative or reduced-intensity conditioning regimens were allowed, depending on the patient age and health status. Of all patients, 14 patients received allo-HSCT during the follow-up period.

### Flow cytometry and cell sorting

Mononuclear cells (MNCs) were isolated from bone marrow aspirates, collected at diagnosis, by density gradient centrifugation (Ficoll-Paque, GE Healthcare Life Sciences). The experiments were done on fresh cells. Primary AML cells were washed and resuspended into 100 μl of cold (4°C) Phosphate Buffered Saline solution (PBS) + 2% fetal calf serum (FCS) and incubated for 30 minutes on ice with combinations of fluorescein isothiocyanate-(FITC), Peridinin-Chlorophyll-Protein Complex-Cy5.5- (PerCP-CY5.5), phycoerythrin- (PE), phycoerythrin-Cy7- (PE-CY7), allophycocyanin (APC)-labeled monoclonal antibodies (MoAbs). Anti-CD45 PerCP-CY5.5, anti-CD19 APC, anti-CD7 FITC, anti-CD33 PE-CY7, anti-CD13 PE, anti-CD10 APC, anti-CD34 FITC, anti-HLA-DR PE-CY7, anti-CD117 PE, anti-cCD3 APC, anti-MPO FITC, anti-cCD79a PE, anti-CD14 APC, anti-CD64 FITC, anti-CD2 PE-CY7, anti-CD11c PE, anti-CD15 FITC, anti-CD56 PE-CY7, anti-CD66c PE, anti-CD34 APC, anti-CD11b FITC, anti-CD38 PE-CY7 and anti-CD123 PE MoAbs were all from BD Biosciences (San Jose, CA). After antibody staining, cells were washed with cold PBS, resuspended in 1 ml of cold PBS and stained for 5 minutes with 2 μg/ml propidium iodide (Sigma-Aldrich), enabling exclusion of dead cells. Cells were kept on ice until fluorescence-activated cell sorting (FACS) analysis. Data acquisition was performed using a FACSAira flow cytometery with BD FacsDiVa software (BD Biosciences, Franklin Lakes, NJ, USA) at the Institute of Hematology, Flow Cytometry Core.

**Figure 1:**
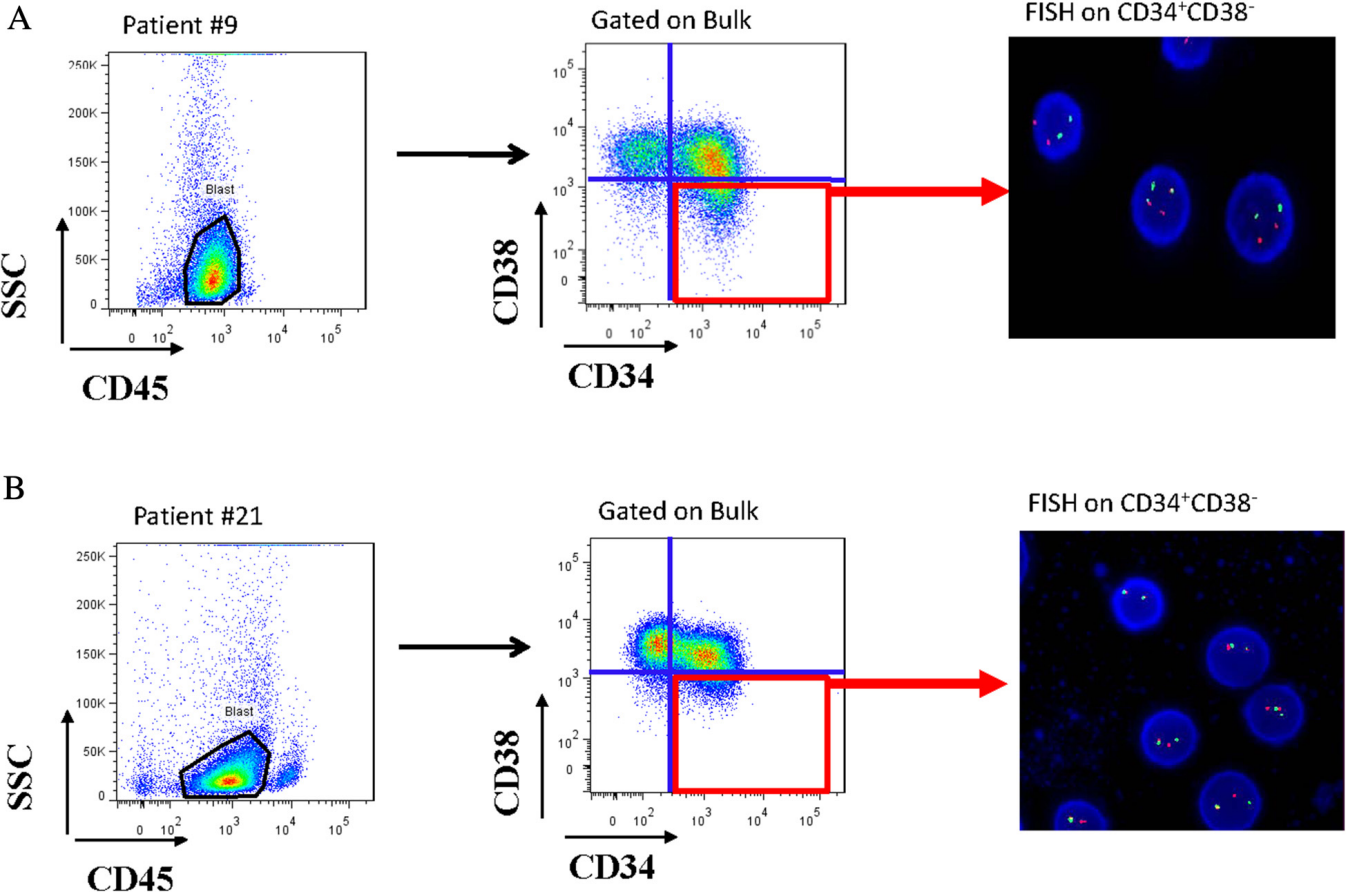
Flow-FISH strategy results for representative patients with AML1/ETO (A) (sample #9) and CBFβ/MYH11 (B) (sample #21) in the blast population are illustrated.

To sort the stem cell subpopulation, a total of 1 × 10^6^ primary AML MNCs was stained with the following conjugated antibodies: monoclonal PE-conjugated anti-CD34, FITC-conjugated anti-CD38 and APC-conjugated anti-CD45 (BD Biosciences). Gates were set to detect the CD34^+^CD38^-^ cells as shown in Figure 1. Cells were sorted using a FACSAria flow cytometery. Cells were kept on ice during the whole procedure. Purity of sorted populations was >99%.

### Flow-fish analysis

As part of the diagnostics for AML, chromosome analyses (G-banding) and FISH were performed on diagnostic BM samples of all AML patients. For Flow-FISH analysis, 1000 to 3000 cells from CD34 + CD38- cell subpopulation were directly sorted into 20 μL drops of PBS that were placed on a grease-free glass slide. The cells were then fixed with 3:1 methanol-glacial acetic acid (Sigma-Aldrich). Air-dried slides were analyzed by FISH in our laboratory, using probes specific for the patient’s known cytogenetic abnormality (Abbot Molecular).

Probes were denatured and hybridized according to manufacturer’s instructions. For evaluation of the Flow-FISH results, hybridization and deletion signals were scored in 500 interphase nuclei with an Axioscop 20 (Carl Zeiss, Jena, Germany) fluorescence microscope with three single-band-pass filters and one triple-band-pass filter. The images were captured with a digital camera using CytoVision 4.1 software (Applied Imaging Corp., Newcastle, U.K.). Results were expressed as percentage FISH-positive cells relative to number of cells analyzed.

### Statistics

Complete response and relapse rates were defined according to the Cheson criteria [14]. Pair-wise comparisons between patients’ characteristics (covariates) were performed using the Mann–Whitney test or Kruskal-Wallis test for continuous variables and with the Fisher’s exact test for categorical variables. Overall survival (OS) was measured from the date of diagnosis until death or last follow-up and events-free survival (EFS) from the date of complete remission (CR) until death, relapse or last follow-up, respectively. Patients alive in complete remission were censored at the time of last contact. Overall and events-free survival rates were estimated by the Kaplan-Meier method and compared using the log-rank test [15]. The cutoff value for FISH^+^CD34^+^CD38^-^ was chosen at a percentage that resulted in the largest difference in survival between the two groups defined by that cutoff. In computations of the cumulative incidence of leukemic relapse (CIR), death and relapse were included as competing events; survival was counted as a censored event. The CIR was compared between groups using the method of Gray, with estimation determined by the method of Kalbfleisch and Prentice [16]. Hazard ratios are given with 95% confidence intervals (95% CI). Survival-time data (events-free survival and overall survival) and covariates (age, leukocyte count, karyotype, *FLT3* mutations and percentage of CD34^+^CD38^-^FISH^+^ cells) were analyzed using the method of backward Cox proportional hazards regression. All calculations were performed using the R 2.7.2 software package.

## Results

Patient characteristics are shown in Table 1. The whole cohort comprised 45 patients with AML with FISH-detectable cytogenetic abnormalities. Distribution of karyotype was provided in Additional file 1: Table S1. After 1 or 2 courses of induction therapy, CR was obtained in 39 patients (86.7%), 2 (4.4%) was not in remission (> 20% blast cells in the bone marrow), and 4 (8.9%) patient died due to febrile neutropenia and sepsis during the course of induction therapy. In the 39 patients achieving CR, 30 (76.9%) were assigned to consolidation therapy consisted of a four- to five-cycle (2 g/m^2^/12 h for 3 days) cytarabine-based chemotherapy, while the rest 9 (23.1%) patients received allogeneic hematopoietic stem cell transplantation after two- or three-cycle cytarabine-based chemotherapy. The median follow-up time for survival was 12 months (range: 1–27). After 24 months, 12 patients (26.7%) had experienced relapse, whereas 7 patients (17.9%) had died due to non-relapse treatment-related complications, 3 from treatment-related complications of transplantation and 4 from infection after chemotherapy (Table 2).

**Table 2:** The treatment outcome according to LIC Level

**Characteristics**	**Low LIC load**	**High LIC load**	**P value**
	**N = 20**	**N = 25**	
Failure of induction therapy	2(10%)	4(16%)	0.678
Overall CR	18(90%)	21(84%)	0.678
Overall relapse	2/18(11.1)	10/21(47.6)	0.018
Treatment-related mortality	2/18(11.1)	5/21(23.8)	0.418
2-year OS	0.7257[0.4082, 0.8916]	0.1675[0.0323, 0.3947]	0.000
2-year EFS	0.6723[0.3262, 0.8687]	0.1633[0.0328, 0.3825]	0.003
2-year CIR	56.7% ± 5.1%	18.0% ± 1.5%	0.021

### LIC assessment by flow-fish

To distinguish normal and leukemic cells within the CD34^+^CD38^-^ cell compartment, we established a unique protocol for conducting FISH on flow-sorted cells. Briefly, CD34^+^CD38^-^ populations from AML patients at diagnosis with FISH-detectable cytogenetic abnormalities were sorted onto glass slides. We could sort as few as 1000 cells on slides, from which at least 500 cells were successfully scored by FISH. Results were calculated as the percentage of FISH-positive cells relative to number of cells analyzed, and the median level was 68.2% (range, 7.8%-89.6%). Results for representative patients (Patient #9 AML1/ETO; Patient #21 CBFβ/MYH11) are illustrated in Figure 1.

### Outcome according to LIC levels

The percentage of leukemia initiating cells (FISH^+^CD34^+^CD38^-^) was then quantified as the value of the percentage of FISH-positive cells multiplies the percentage of CD34^+^CD38^-^cells in the blast (CD45^dim^SSC^low^). The percentage of FISH^+^CD34^+^CD38^-^ cells was evaluated in 45 primary AML samples at diagnosis. The median expression of CD34^+^CD38^-^ was 3.3% in the blast (range, 0.1–52.8%). The FISH^+^CD34^+^CD38^-^ cells were rare in the newly diagnosed AML patients, constituting a median of only 2.31% (n = 45, range, 0.01%-29.4%) of the total blast cells. According to the LIC level detected by Flow-FISH analysis, patients were categorized into the two groups: low LIC (<1%) and high LIC (≥1%), representing 44.4% and 55.6% of all patients, respectively. The clinical characteristics of the patients are provided in Table 1. Comparison of clinical and laboratory characteristics showed no significant differences between two groups in age, gender, white blood cell (WBC) count, platelet (PLT) count, blast percentage, FAB subtype distribution, *FLT3* mutation status and cytogenetics risk at diagnosis (Table 1).

**Figure 2:**
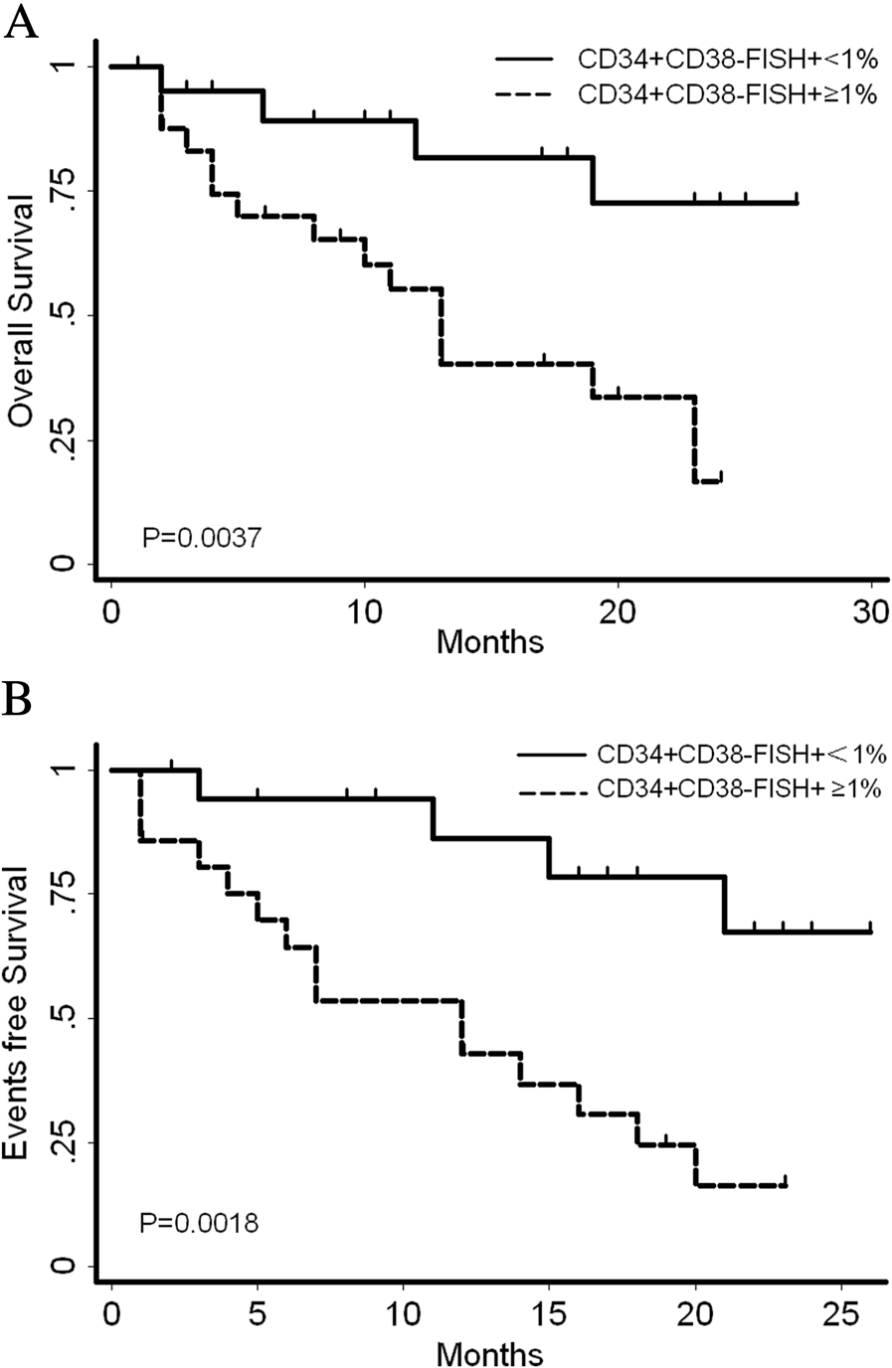
**Comparison of overall survival (A) and events-free survival (B) of AML patients according to FISH + CD34 + CD38- percentage.** A FISH^+^CD34^+^CD38^-^ percentage of over 1% was found to be significantly associated with decreased OS and EFS.

To determine the prognostic impact of detecting LIC load at diagnosis, we analyzed the treatment outcome for 45 patients enrolled in a standardized treatment program (Table 2). The 2-year overall survival for patients with low LIC load was 0.7257[0.4082, 0.8916], compared with 0.1675[0.0323, 0.3947] for the patients with high LIC load (*P* = 0.0037). And 2-year events-free survival were 0.6723[0.3262, 0.8687] versus 0.1633[0.0328, 0.3825], respectively (*P* =0 .0018) (Figure 2).

**Figure 3:**
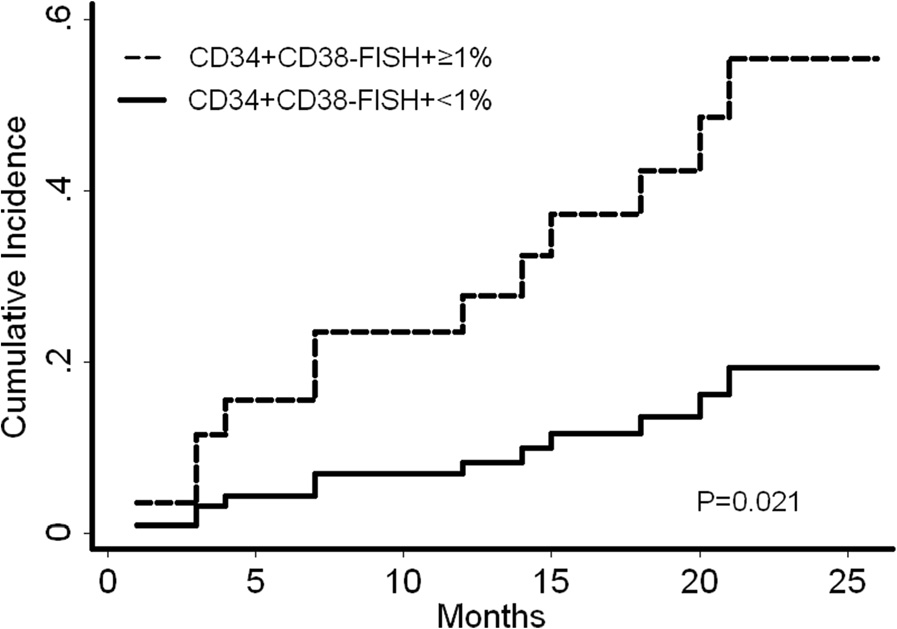
**Cumulative incidence of relapse.** A FISH^+^CD34^+^CD38^-^ percentage of over 1% was found to be significantly associated with higher CIR.

Events, including relapse and death, were more frequent in patients with a high LIC load. Among 21 patients who received CR with a high LIC load, 10 (46.7%) experienced a relapse; among 18 patients who achieved CR with a low LIC load, 2 (11.1%) experienced relapse (*P* = 0.018). 5 treatment related death (TRD) (23.8%) occurred in the 21 patients who achieved CR with a high LIC load, whereas 2 TRD (5.6%) occurred in the 18 patients who received CR with a low LIC load (*P* = 0.418). The estimated 2-year cumulative incidence of leukemic relapse for patients with high LIC load was 56.7% ± 5.1% versus 18.0% ± 1.5% for patients with low LIC load (*P* = 0.021) (Figure 3).

### Multivariate analysis of the prognostic value of flow-fish

In a univariate analysis of recognized prognostic factors, including age, *FLT3* mutation status, leukocyte count, cytogenetics and allo-HSCT, cytogenetics were found to be significantly associated with overall survival (*P* = 0.01), whereas the leukocyte count, age, karyotype, *FLT3* mutation status and allo-HSCT had no impact on events-free survival. We next performed a multivariate Cox proportional hazards model for OS and EFS, a percentage of LIC load of over 1% at diagnosis remained the significant predictor of outcome (*P* = 0.003 for OS; *P* = 0.004 for EFS) (Tables 3 and 4).

**Table 3:** Analysis of covariates associated with overall survival

	**Univariate analysis**		**Cox regression**	
	***P***	**HR**	***P***	**HR**
		**95% CI**		**95% CI**
Age	0.085	2.176[0.898, 5.276]	0.562	1.393[0.454, 4.278]
FLT3 mutation	0.363	0.486[0.103, 2.297]	0.644	0.639[0.096, 4.275]
WBC ≥ 50 × 10^9^	0.250	1.763[0.670, 4.636]	0.105	2.844[0.802, 10.085]
Cytogenetics	0.010	2.046[1.190, 3.519]	0.068	1.914[0.953, 3.846]
Allo-HSCT	0.228	1.810[0.689, 4.754]	0.015	4.581[1.338, 15.684]
FISH + CD34 + CD38- ≥ 1%	0.009	4.319[1.436, 12.987]	0.003	7.048[1.918, 25.899]

*P* of the univariate analysis is the *P* value of the log rank test. HR is the value of the hazard ratio. 95% CI is the 95% confidence interval of the hazard ratio. Data from 45 patients were complete and were included in the Cox proportional-hazards regression.

**Table 4:** Analysis of covariates associated with events-free survival

	**Univariate analysis**		**Cox regression**	
	***P***	**HR**	***P***	**HR**
		**95% CI**		**95% CI**
Age	0.473	1.428[0.540, 3.780]	0.914	0.933[0.265, 3.287]
FLT3 mutation	0.109	0.267[0.053, 1.340]	0.310	0.375[0.056, 2.494]
WBC ≥ 50 × 10^9^	0.466	1.469[0.523, 4.131]	0.417	1.645[0.494, 5.484]
Cytogenetics	0.100	1.620[0.912, 2.875]	0.097	1.854[0.893, 3.847]
Allo-HSCT	0.508	1.371[0.538, 3.496]	0.092	2.674[0.853, 8.389]
FISH + CD34 + CD38- ≥ 1%	0.005	4.955[1.621, 15.148]	0.004	6.354[1.806, 22.356]

*P* of the univariate analysis is the *P* value of the log rank test. HR is the value of the hazard ratio. 95% CI is the 95% confidence interval of the hazard ratio. Data from 39 patients were complete and were included in the Cox proportional-hazards regression.

## Discussion

AML is a highly heterogeneous disease from the biological and clinical standpoint, for which prognostic factors have become increasingly important in the choice and planning of therapeutic procedures. Currently, cytogenetic and molecular aberrations are the best prognostic indicators for AML patients [17]. However, these factors predict primarily for groups of patients and cannot prognosticate well for individual patients within any given risk strata. For example, CBF cytogenetic abnormalities are generally considered favorable; yet approximately half of these patients relapse [18-20]. So, new prognostic tools based on biological analyses need to be developed.

AML is regarded as a stem cell disease, and the failure of chemotherapy in eradicating primitive leukemia initiating cells likely contributes to the relapse of AML. Numerous studies have emphasized the correlation between the enrichment of the CD34^+^CD38^-^ phenotype in AML at diagnosis and a high level of residual disease after treatment [12,13]. And it is generally accepted that CD34^+^CD38^-^ cells are enriched for LICs, however, this population is heterogeneous and includes both normal and leukemic stem cells. In this regard, measure of “real” LICs promises to serve as a powerful, individualized prognostic tool, enhancing delivery of risk-adapted therapies. Recent data have generated important advances in the field, including the identification of novel leukemia initiating cells-specific cell surface antigens. Several studies have showed that the aberrant expression of specific markers on the cell surface of leukemia progenitors is characteristic for LIC, differentiating them from their normal counterparts, including CD90, CD123, CLL-1, CD96, CD47, Tim3 and so on [8-11]. However, aberrant marker-positive CD34^+^CD38^-^ cells at diagnosis are partly of malignant origin, while the corresponding marker-negative population is not totally of normal origin. For example, Rhenen et al. reported that CD123 seems not very useful in leukemic stem cell detection due to the high expression of CD123 in normal control samples [21].

In the present study, we established the Flow-FISH protocol as an effective method to distinguish normal and leukemic cells within the CD34^+^CD38^-^ cell subpopulation. As leukemic cytogenetic marker was never present in normal counterparts, we could get the reliable result of “real” LIC load in leukemic burden through Flow-FISH analysis for patients with recurrent cytogenetic abnormalities. We showed that this leukemia initiating cells subpopulation was not so rare in patients, and we found that FISH^+^CD34^+^CD38^-^ cells ranged from 0.01% to 52.8% in the bulk of blasts. Although the results of the present finding need to be confirmed in a larger cohort, our data do show that a high LIC load at diagnosis determines a high relapse rate of leukemia and a poor clinical outcome. A percentage of FISH^+^CD34^+^CD38^-^ cells of more than 1% is strongly associated with reduced OS and EFS suggesting that residual disease is likely to be high in these patients. Hence, the assessment of the level of FISH^+^CD34^+^CD38^-^ cells at diagnosis could help clinicians to identify patients as an effective prognostic indicator in AML with recurrent chromosomal abnormalities, eg. CBF-AML (Data not shown), and thereby refining the selection of therapeutic strategies and, possibly, long-term clinical outcome.

This ability to distinguish these cell populations offers the potential to simultaneously observe the efficacy of chemotherapy drugs against putative LICs and their toxicity against normal HSCs. It may also facilitate better identification of therapeutic targets. Recently, Minderman et al. reported a unique technology to perform FISH in suspension [22]. The ability to visualize and perform photometric/morphometric analysis of imagery from tens of thousands of cells thus combines quantitative image analysis with the statistical power of flow cytometry, may further enhance the sensitivity and specificity of LIC detection.

To the best of our knowledge, Flow-FISH is used, for the first time, to detect LIC subpopulation in human bone marrow samples. In accordance with our results, high levels of “real” LIC load detected at diagnosis in AML patients treated with standard chemotherapy, was significantly correlated with increased risk of poor clinical outcome. However, it is still not clear if the prognostic value of the LIC load detected with Flow-FISH is associated with the risk stratification of the associated cytogenetic abnormality and other known gene mutations such as FLT3-ITD because, most probably, of the limited cases in this cohort. Future larger cohort studies should solve this issue. In conclusion, Flow-FISH discrimination of leukemic and normal candidate stem cells could be used as a powerful tool to predict clinical outcome and help physicians to evaluate criteria for treatment in AML with recurrent cytogenetic abnormalities.

## Findings

Flow-FISH protocol was a useful method to distinguish normal and “real” leukemic initiating cells within the CD34^+^CD38^-^ cell subpopulation. And high LIC Load at diagnosis was significantly correlated with increased risk of poor clinical outcome.

## Competing interests

The authors declare that they have no competing interests.

## Authors’ contributions

*LW* collected and verified patient information, analyzed and interpreted data, and wrote the manuscript; *LG* analyzed data, performed statistical analysis and verified patient information; *SX* and *ML* performed cell sorting and MRD monitoring by flow cytometry; *SG* and *HQ* performed FISH; *HQ, XX, XN, LC, SL, JC, XS, WZ, and JY* performed diagnosis and treatment for patients; *JW* and *XH* designed research, interpreted data, and critically reviewed the manuscript. All authors read and approved the final manuscript.

## Supplementary Material

Additional file 1 **Table S1.** FISH-detectable cytogenetic abnormalities of the 45 patients included in this study. Click here for file
