# Supplementary material for: FISH+CD34+CD38- cells detected in newly diagnosed acute myeloid leukemia patients can predict the clinical outcome
Source: J Hematol Oncol. 2013 Nov 7;6:85. doi: 10.1186/1756-8722-6-85 (PMC4028871; doi:10.1186/1756-8722-6-85)
Supplement: Additional file 1 — Table S1. FISH-detectable cytogenetic abnormalities of the 45 patients included in this study. [file 1756-8722-6-85-S1.docx]

**Additional file**

**Table S1. FISH-detectable cytogenetic abnormalities of the 45 patients included in this study.**

| **FISH-detectable cytogenetic abnormalities** | **N(%)** |
| --- | --- |
| **t(8;21)*** | 22(48.9) |
| **Inv(16)**§ | 6(13.3) |
| **t(11;19)** | 3(6.7) |
| **Other 11q23** | 2(4.4) |
| **t(6;9)** | 1(2.2) |
| **7q-** | 2(4.4) |
| **9q-** | 2(4.4) |
| **Trisomy 8﹟** | 5(11.1) |
| **Trisomy 21** | 2(4.4) |

* 3 cases with complex karyotypes [＞3 abnormalities and include t(8;21)];

§1 case with complex karyotypes [＞3 abnormalities and include inv(16)];

﹟2 case with complex karyotypes [＞3 abnormalities and include +8]
